# Inter-annual variability patterns of reef cryptobiota in the central Red Sea across a shelf gradient

**DOI:** 10.1038/s41598-022-21304-2

**Published:** 2022-10-09

**Authors:** R. Villalobos, E. Aylagas, J. K. Pearman, J. Curdia, D. Lozano-Cortés, D. J. Coker, B. Jones, M. L. Berumen, S. Carvalho

**Affiliations:** 1 King Abdullah University of Science and Technology (KAUST), Red Sea Research Center, Thuwal, 23955-6900 Saudi Arabia; 2 The Red Sea Development Company, 5th Floor, MU04 Tower, ITCC Complex, AlRaidah Digital City, Al Nakhil District 3807, Riyadh, 12382-6726 Saudi Arabia; 3 Coastal and Freshwater Group, Cawthron Institute, Nelson, New Zealand; 4 Environmental Protection, Saudi Aramco, Dhahran, Saudi Arabia

**Keywords:** Biodiversity, Community ecology, Marine biology

## Abstract

The combination of molecular tools, standard surveying techniques, and long-term monitoring programs are relevant to understanding environmental and ecological changes in coral reef communities. Here we studied temporal variability in cryptobenthic coral reef communities across the continental shelf in the central Red Sea spanning 6 years (three sampling periods: 2013–2019) and including the 2015 mass bleaching event. We used a combination of molecular tools (barcoding and metabarcoding) to assess communities on Autonomous Reef Monitoring Structures (ARMS) as a standardized sampling approach. Community composition associated with ARMS for both methodologies (barcoding and metabarcoding) was statistically different across reefs (shelf position) and time periods. The partition of beta diversity showed a higher turnover and lower nestedness between pre-bleaching and post-bleaching samples than between the two post-bleaching periods, revealing a community shift from the bleaching event. However, a slight return to the pre-bleaching community composition was observed in 2019 suggesting a recovery trajectory. Given the predictions of decreasing time between bleaching events, it is concerning that cryptobenthic communities may not fully recover and communities with new characteristics will emerge. We observed a high turnover among reefs for all time periods, implying a homogenization of the cryptobiome did not occur across the cross shelf following the 2015 bleaching event. It is possible that dispersal limitations and the distinct environmental and benthic structures present across the shelf maintained the heterogeneity in communities among reefs. This study has to the best of our knowledge presented for the first time a temporal aspect into the analysis of ARMS cryptobenthic coral reef communities and encompasses a bleaching event. We show that these structures can detect cryptic changes associated with reef degradation and provides support for these being used as long-term monitoring tools.

## Introduction

Coral reefs harbor approximate 35% of the total marine biodiversity^1^, and recent estimates indicate that 830,000 multicellular species inhabit coral reefs^2^. However, it is estimated that 85–99% of coral reef species are still to be described; a large proportion is expected to be discovered in the small inconspicuous organisms such as cryptobenthic fishes^3^ and invertebrates^4^, which inhabit cracks and crevices provided by the reef framework^5^. These organisms constitute the reef cryptobiome^5^ and due to their characteristics (small size, cryptic behavior, and color patterns) have regularly been overlooked in reef surveys. However, the highly specious cryptobiome is crucial for coral reef dynamics. They are an essential food source for predators^6,7^ and play an important role cycling nutrients in the reef food web^8^.

To establish strategies to halt or slow down the current trend in species loss, first we need to catalog biodiversity through collections and descriptions of these organisms^1,9^. This is particularly relevant in areas where comparatively limited research has been conducted, such as the Red Sea^10^. In addition, a shortage of taxonomists is creating a bottleneck in the description of new species^11^. Recent advances in molecular techniques have improved our ability to separate organisms’ identity based on regions in their DNA code into “operational taxonomic units”, fostering more comprehensive assessments over space and time in species-rich areas^12^. DNA-based techniques have an additional advantage in extracting the identifications of larvae or immature organisms, an even greater challenge for morphological-based identifications^13^. Therefore, studies using DNA-based techniques are becoming increasingly used in coral reef ecosystems^5,14–19^. Recent studies using eDNA in the Red Sea and elsewhere have detect conspicuous and cryptobenthic reef species^20,21^, proved suitable for monitoring multitrophic community variations^22^ and estimate levels of anthropogenic pressure^23^.

Recent reports have linked biodiversity loss with impairment of the ocean’s ability to deliver goods (e.g., food provision) and services (e.g., maintenance of water quality)^24^, negatively affecting human well-being. Coral reefs, in particular, are known to support the livelihoods of more than 500 million people in the world and generating US$35.8 billion dollars annually^25^. A sound understanding of how species change in space and time, and the underlying processes driving those changes is critical for elucidating the relationship between habitat degradation and biodiversity^26^. Global pressures, such as increasing sea water temperature as a result of elevated CO_2_ levels, have been linked to a higher intensity and frequency of bleaching events worldwide^27^, resulting in dramatic coral loss, and potentially a relative dominance of rubble^28^. Scleractinian corals along with crustose coralline algae contribute to reef building, with a critical role in maintaining reef structure and consequently reef biodiversity^29,30^. The shift to reefs dominated by non-calcifying organisms and an increase in non-framework building corals^31–34^ conveys an immediate decline in species with obligate relationships with corals, followed by an alteration in the community of fishes and invertebrates^35,36^. Maintaining biodiversity is crucial for securing the stability of ecosystems’ functioning^37,38^ and, consequently, the conservation of coral reefs and their associated biodiversity, which is a fundamental step to ensure the delivery of critical goods and services^39^.

Long-term studies provide unique information regarding environmental and ecological changes over time^40,41^. Time series provide fundamental information about natural variability and are key to set critical thresholds above which significant and undesirable changes may be detected. Ultimately, these thresholds will be fundamental in the early detection of ecosystem degradation. Regrettably, funding for sustaining long-term monitoring projects is decreasing over recent years^41^. Marine ecosystems are subject to natural variability in physico-chemical (e.g., temperature, salinity, light availability) and biological forcing factors (e.g., competition, predation, food quality and quantity), driving the spatio-temporal dynamics of biological communities^40,42–44^. It is important to understand the natural temporal variances in biological communities and their driving factors, so that the effects of human induced disturbances can be detected, assessed, and modeling of future trajectories predicted^45,46^. To the best of our knowledge, efforts in documenting biodiversity patterns of the cryptobiome and its ecological drivers following standardized approaches across a temporal time scale are lacking in the Red Sea and are very limited worldwide.

The application of standardize methods to address a specific question is paramount to be able to scale up findings from local to global scales^14^. One example is the Autonomous Reef Monitoring Structures (ARMS) to investigate changes in the cryptobiome. In response to the need of finding standardized tools to investigate patterns in space and time of this overlooked biological reef component, during the Census of Marine Life (CoML), a team of scientists developed the Autonomous Reef Monitoring Structures. Each ARMS unit consists of nine layers of square PVC plates (22.5 cm by 22.5 cm), mimicking the structural complexity of the reef environment with multiple ecological niches represented through different levels of exposure to light and water flow. They are usually deployed in a coral reef between one and three years to allow a mature community of the cryptobiome to settle^17,47^. The removal and re-deployment allows for sustained long-term observations of biodiversity to be achieved. ARMS have been attracting the attention of the scientific community, with hundreds of deployments across the Indo-Pacific, Red Sea, Atlantic, Mediterranean, Black and Baltic Seas in different benthic habitats^14,48–54^. The relevance and potential of this tool to scaling up spatial and temporal trends has even resulted in the establishment of a molecular research network in Europe^55^.

In this study, we investigate patterns of cryptobenthic coral reef communities at three time periods (2015, 2017 and 2019) across a shelf gradient in the central Saudi Arabian Red Sea. During this 6-year period (2013: first deployment of ARMS), reefs in this region experienced the global mass bleaching event in 2015/2016, giving us the opportunity to investigate not only the spatio-temporal changes in cryptobenthic biodiversity across a shelf gradient but also to analyze the potential response of the cryptobiome to this bleaching event. We hypothesize that shelf position will be a driver of biodiversity patterns^16^ and these patterns will be maintained through time. Considering that the effects of bleaching tend to attenuate with distance from shore^28^, we also hypothesize that temporal turnover will be higher in nearshore than offshore reefs in response to a bleaching event, as more changes are expected in the nearshore benthic coral reef structure.

## Results

### Alpha diversity

**Figure 1:**
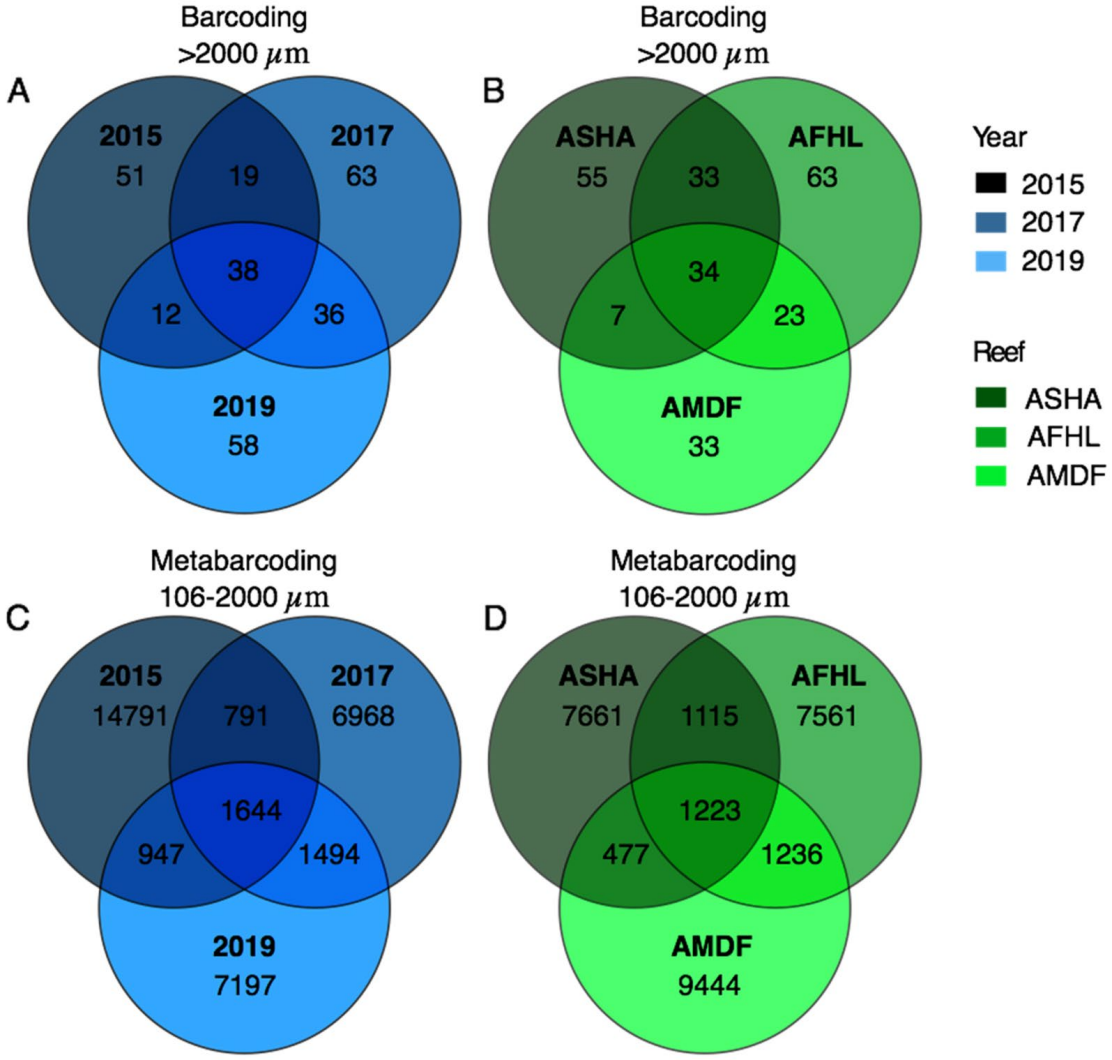
Venn diagrams showing the number of OTUs of the > 2000 µm fraction unique to each time (**A**) and reef (**B**) and the shared between them. (**C**) and (**D**) show the number of ASVs of the metabarcoded 106–2000 µm fraction unique and shared between time (**C**) and reef (**D**). ASHA stands for Abu Shoosha reef, AFHL for Al Fahal, and AMDF for Abu Madafi. 2015, 2017, and 2019 are the dates of the recovery of the ARMS and correspond to the deployment periods of 2013–2015, 2015–2017 and 2017–2019, respectively.

A total of 277 operational taxonomic units (OTU) and 1,723 specimens were identified from the > 2000 µm size range of the 33 ARMS units. Most OTUs were present at a single time or reef. However, we were unable to retrieve ARMS from Abu Shootaf in 2019. Over the three sampling periods only 38 OTUs (14%) were identified in all three periods. The highest number of OTUs (74 OTUs; 27%) were shared between 2017 and 2019. In contrast, 2015 and 2019 shared the fewest number of OTUs (50 OTUs; 12%). This pattern between sampling times was observed for most reefs, except Al Fahal (Fig. S-1). Only 34 OTUs were shared across the three reefs (14%) (Fig. 1). Abu Madafi, the offshore reef, shared a lower proportion of OTUs with the nearshore reef (Abu Shoosha; 41 OTUs 17%) compared with the midshore reef (Al Fahal; 57 OTUs 23%). These patterns were consistent through sampling times when analyzing each reef separately (Fig. S-1). Kruskal–Wallis showed that the number of OTUs (Chi Square = 6.7, *p* = 0.04) and the overall abundance (Chi Square = 10.2, *p* < 0.01) was significantly different between sampling times (Table S-1). Average number of OTUs per ARMS increased from 19 OTUs, in 2015, to approximately 30 both in 2017 and 2019. The lowest number of individuals was also observed in 2015 (~ 28 organisms per ARMS) and peaked in 2017 (~ 86 organisms per ARMS), before declining to ~ 43 organisms per ARMS in 2019 (Table S-1). The Shannon diversity index was equivalent between sampling times and reefs with no significant differences detected (Table S-1).

In the 106–2000 µm metabarcoding fraction 33,832 amplicon sequence variants (ASV) were obtained from the 33 ARMS units. Most of the ASVs were present in a single sampling period (86%, 28,966 ASVs) or reef (86%, 24,666 ASVs) (Fig. 1). Almost half of the ASVs were present exclusively in 2015 (44%, 14,791 ASVs). The 2015 sampling period had the highest number of unique ASVs when observing each reef (Fig. S-2). Only 5% (1644 ASVs) were persistent through the study period and 4% (1223 ASVs) present in all the reefs. The 2017 and 2019 sampling times shared 9% of the total ASVs (3138 ASVs), 2015 and 2017 shared 7% (2435 ASVs), and 2015 and 2019 8% (2,591 ASVs). Similarly, to the larger barcoded fraction the Abu Madafi reef shared fewer ASVs with the nearshore reef Abu Shoosha (1700 ASVs; 6%) than with Al Fahal (2459 ASVs; 9%). This pattern is observed in all the sampling times (Fig. S-2).

The number of ASVs in the metabarcoding fraction (106–2000 µm) was significantly different between sampling times (Chi Square = 7.8, *p* = 0.02) but not between reefs (Chi Square = 1.7, *p* = 0.42). Unlike the observed for the barcoded fraction, the average number of ASVs per ARMS was lowest in 2017 (1618) and highest in 2019 (2139), close to the number of ASVs observed in 2015 (2072).

**Figure 2:**
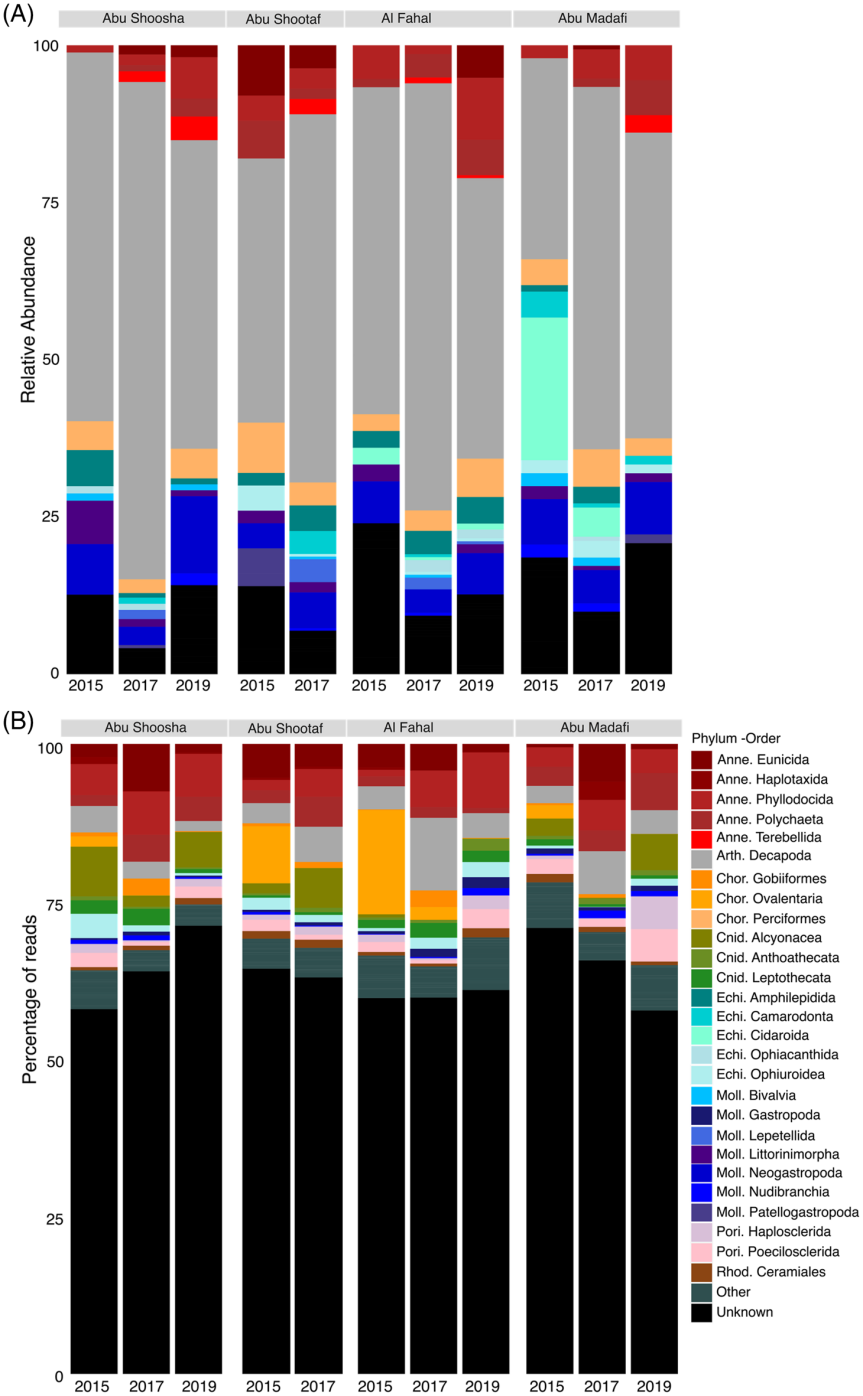
Relative abundance of each Order per reef as an average of the 3 units of the Barcoding fraction (**A**) and relative abundance of reads for each Order of the metabarcoding fraction (**B**). The orders assigned to the group ‘Other’ contribute to 5% of the total abundance.

For both, the barcoding and the metabarcoding fractions, Annelida increased in relative abundance in 2017 (Fig. 2). The barcoding fraction was dominated by Decapoda (Arthropoda) with 60% of the total specimens. And, the metabarcoding fraction was dominated by Annelida (18%), Arthropoda (27%), Chordata (8%), and Cnidaria (6%).

### Beta-diversity

**Figure 3:**
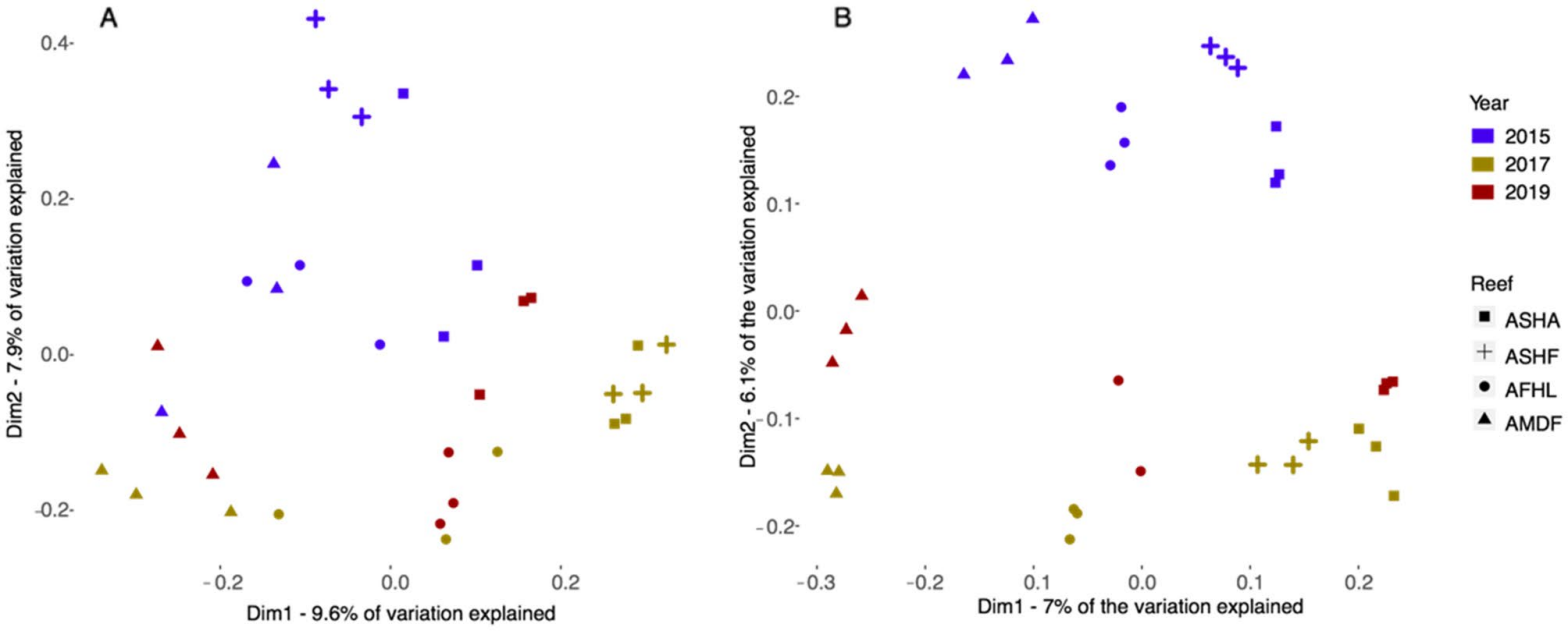
Principal coordinate analysis of the (**A**) barcoding > 2000 µm and (**B**) metabarcoding 106–2000 µm fractions using the presence absence matrix and the Jaccard dissimilarity metric to assess differences in the community composition between reefs and time. Reef names are abbreviated as: *ASHA* Abu Shoosha, *ASHF* Abu Shootaf, *AFHL* Al Fahal, and *AMDF* Abu Madafi.

In the > 2000 µm fraction there was a significant interaction between reef and time (permutational multivariate analysis of variance (PERMANOVA) *p* < 0.01; Table S-2) for both Jaccard and Bray–Curtis dissimilarities. We found significant differences among sampling times in the post-hoc test of both matrices (Tables S-2 and S-3). Overall, the 2015 samples grouped separately from those collected in 2017 and 2019 along the second axis of the PCO that explained 7.9% of the variation (Fig. 3A). And, the first axis differentiated the reefs. There was a general trend for samples from the same reefs in 2015 clustering closer to their counterparts from 2019 than 2017, suggesting a higher similarity between the first and the last sampling period, than between the first and the second period (Fig. 3A and B). The cross-shelf gradient was mainly maintained within each sampling period, with higher similarity among the communities across the shelf in 2015 (for the 106–2000 µm fraction), becoming more dissimilar after the bleaching event (i.e., samples from different reefs in 2017 and 2019 are farther apart in the plot). Reefs were significantly different from each other except Abu Shoosha and Al Fahal as revealed in the post-hoc test of the PERMANOVA (Table S-3). A similar trend was observed for the metabarcoded fraction (106–2000 µm) with a significant interaction (PERMANOVA *p* < 0.01; Table S-2) for both Jaccard and Bray–Curtis. Significant differences between all sampling times and between all reefs were also observed (Table S-3).

**Figure 4:**
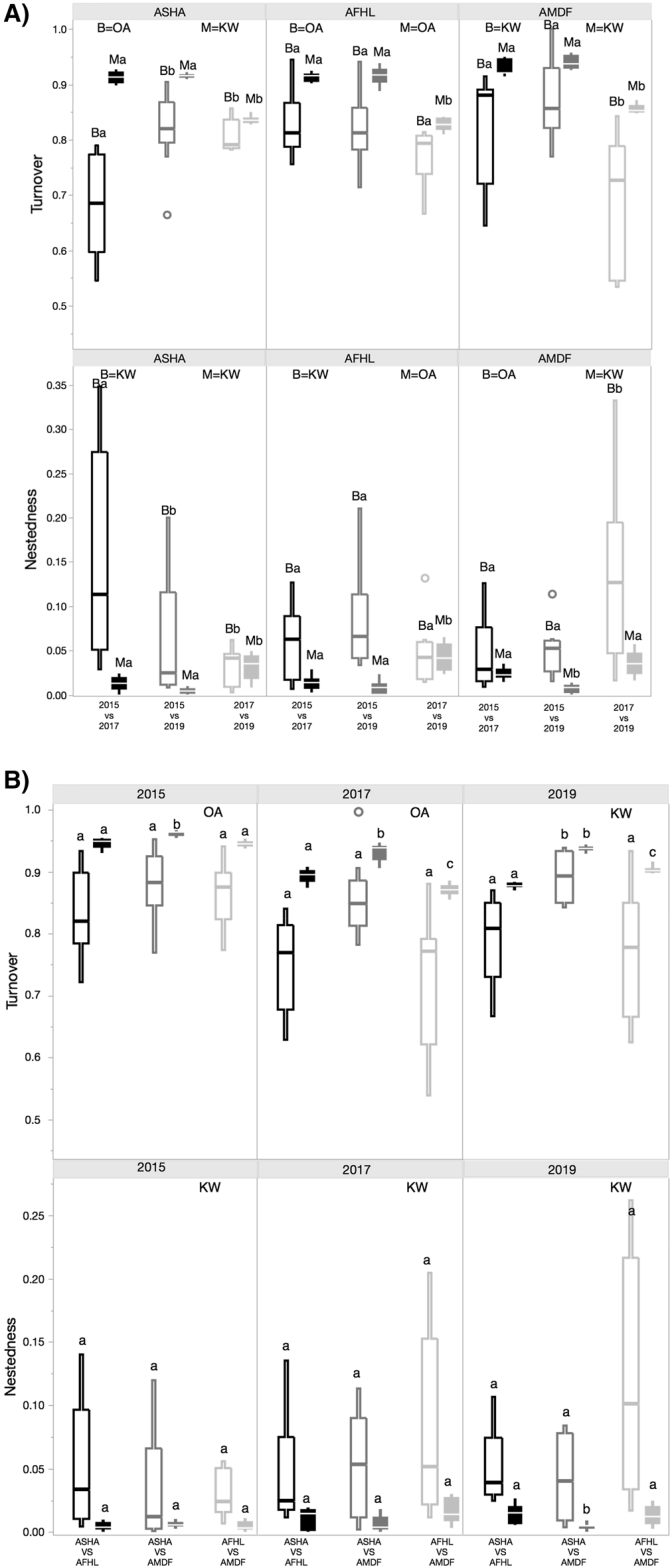
Turnover and nestedness through time and in the cross-shelf. (**A**) Comparisons between pairs of sampling times within the same reef in turnover [top graphs in (**A**) and nestedness [bottom graphs in (**A**)] of the metabarcoding and barcoding fractions for the Jaccard dissimilarity metric using presence absence data. The arrangement of figure A is made to appreciate the distinctions in turnover and nestedness through time in each reef. (**B**) Turnover and nestedness between pair of reefs in the cross-shelf for each sampling date using the presence absence data for the Jaccard dissimilarity metric. Samples from the barcoding fraction (> 2000 µm) are delineated and from the metabarcoding fraction (106–2000 µm) filled. Lower case a and b letter marks the distinction of groups obtained from one-way ANOVA (OA) or from Kruskal–Wallis (KW). *Reef names are abbreviated as: ASHA* Abu Shoosha, *ASHF* Abu Shootaf, *AFHL* Al Fahal, and *AMDF* Abu Madafi.

For both the barcoding and metabarcoding datasets, turnover was a higher component of beta diversity than nestedness. For the barcoding dataset, different patterns were observed for turnover and nestedness temporally at the reef level. At the offshore reef Abu Madafi, turnover (*p* < 0.01; Fig. 4A) and nestedness (*p* = 0.02; Fig. 4A) were significantly lower in 2017–2019 while for the nearshore reef, Abu Shoosha, it was the period 2015–2017 showing significantly lower values (turnover *p* < 0.01; nestedness *p* = 0.03; Fig. 4A), when compared to the other pairs of sampling periods. There were no significant differences in turnover and nestedness between sampling periods in Al Fahal (midshore reef). Comparisons amongst reefs within years showed that the only significant difference in turnover was in 2019 between Abu Shoosha and Abu Madafi (*p* < 0.01; Fig. 4B). There were no significant differences in nestedness between sampling dates or reefs.

In the metabarcoding dataset the only significant temporal differences in turnover—observed in all reefs occurred between 2017 and 2019 (*p* < 0.01 in all reefs; Fig. 4A), where turnover was significantly lower than for the remaining comparisons. However, while nestedness was higher for the 2017–2019 comparison in Abu Shoosha (*p* < 0.01) and Al Fahal (*p* < 0.01), this pattern was not observed in Abu Madafi where the 2015–2017 comparison was significantly lower (*p* < 0.01). Comparisons amongst reefs showed that in 2017 and 2019 all reefs had a significantly different turnover (2017 *p* < 0.01; 2019 *p* < 0.01; Fig. 4B) while in 2015 only the comparison between Abu Madafi and Abu Shoosha was significantly different (*p* < 0.01). In terms of nestedness the only significant difference was observed in 2019 between Abu Madafi and Abu Shoosha (*p* = 0.01).

### Spatio-temporal indicators

**Figure 5:**
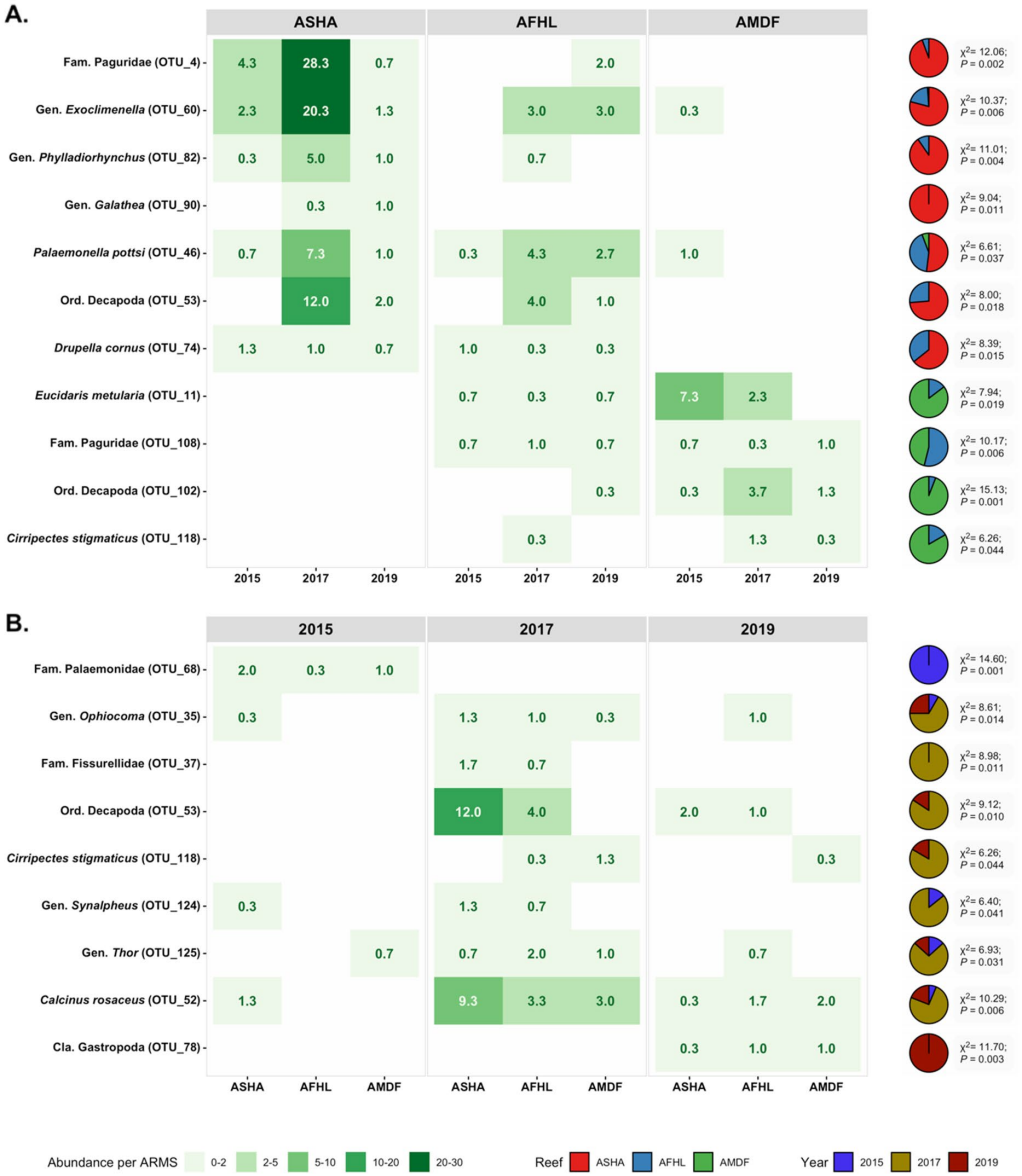
Average abundances per ARMS of the indicator taxa observed for the barcoding fraction by reef (**A**) and sampling year (**B**). Kruskal–Wallis test results are shown in the right side. *ASHA* Abu Shoosha reef, *AFHL* Al Fahal, *AMDF* Abu Madafi.

We identified 18 OTUs with a significant contribution to the differences in spatio-temporal patterns (Fig. 5). Two hermit crabs (Paguridae OTU_4 and OTU_108), two shrimps (*Exoclimenella* OTU_60 and *Palaemonella pottsi* OTU_46), two squat lobsters (*Galathea* OTU_90, *Phylladiorhynchus* OTU_82), two decapods (Decapoda OTU_53 and OTU_102), one gastropod (*Duprella* cornus OTU_74), one blenny (*Cirripectes stigmaticus* OTU_118), and one sea urchin (*Eucidaris metularia* OTU_11) were indicator taxa for the reef factor. Seven indicator OTUs (OTU_4, OTU_60, OTU_82, OTU_90, OTU_46, OTU_53, and OTU_74) had an inshore to offshore gradient in abundance with higher abundance in the inshore reef Abu Shoosha (Fig. 5). And, OTU_11, OTU_102, and OTU_118 presented the opposite trend. Three shrimps (Palaemonidae OTU_68, *Synalpheus* OTU_124, and *Thor* OTU_125), one brittle star (*Ophiocoma* OTU_35), two gastropods (Fissurellidae OTU_37 and Gastropoda OTU_78), one decapod (Decapoda OTU_53), one blenny (*Cirripectes stigmaticus* OTU_118), and one hermit crab (*Calcinus rosaceus* OTU_52) were indicator taxa for the factor time. Seven indicator OTUs (OTU_35, OTU_37, OTU_53, OTU_118, OTU_124, OTU_125, and OTU_52) gained abundance in 2017, and, OTU_78 in 2019. OTU_68 showed the opposite pattern with highest abundance in 2015. When looking at the abundance of the barcoding fraction, the factor reef and time had a significant association with the abundance of the indicator taxa for the reef and time factor (Fig. 5).

**Figure 6:**
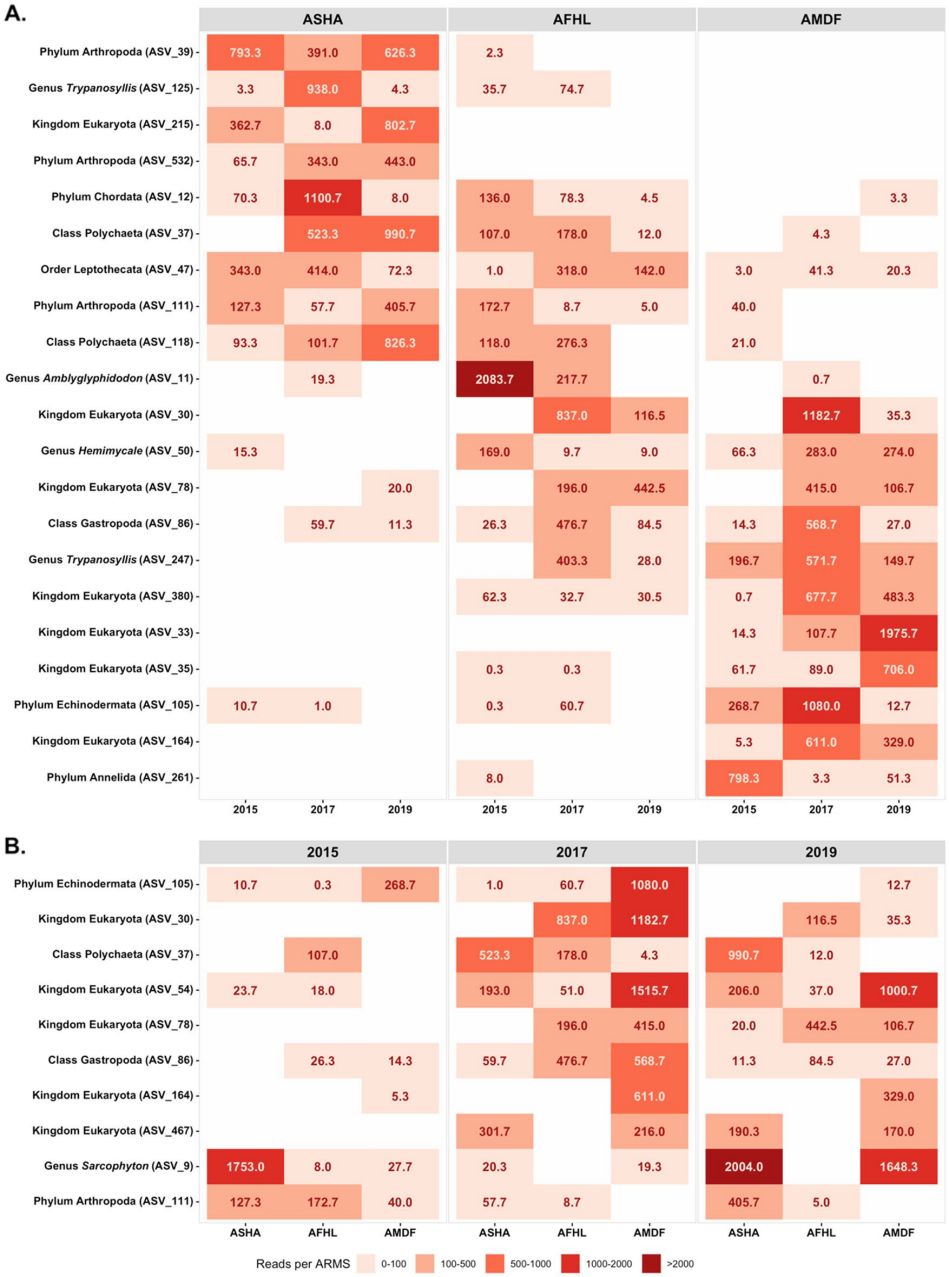
Average number of reads per ARMS of the indicator taxa observed for the metabarcoding fraction by reef (**A**) and sampling year (**B**). *ASHA* Abu Shoosha reef, *AFHL* Al Fahal, *AMDF* Abu Madafi.

We identified 21 ASVs with a significant contribution to the differences in spatial patterns and 10 ASVs in spatial patterns (Fig. 6). Five annelids (*Trypanosyllis* ASV_125 and ASV_247, Annelida ASV_261, and Polychaeta ASV_37 and ASV_118), three arthropods (Arthropoda ASV_39, ASV_532, and ASV_111), two chordates (Chordata ASV_12 and *Amblyglyphidodon* ASV_11), one cnidarian (Leptothecata ASV_47), one echinoderm (Echinodermata ASV_105), one sponge (*Hemimycale* ASV_50), and seven identified only at kingdom level (Eukaryota ASV_215, ASV_30, ASV_78, ASV_380, ASV_33, ASV_35, ASV_164) were indicator taxa for the reef factor. Nine indicator ASVs (ASV_39, ASV_125, ASV_215, ASV_532, ASV_12, ASV_37, ASV_47, ASV_111, ASV_118) presented an inshore to offshore gradient with higher abundance in the inshore reef Abu Shoosha and eleven indicator ASVs (ASV_30, ASV_50, ASV_78, ASV_86, ASV_247, ASV_380, ASV_33, ASV_35, ASV_105, ASV_164, ASV_261) showed the opposite pattern with higher abundance in the offshore reef Abu Madafi. One annelid (Polychaeta ASV_37), one Arthropoda (Arthropoda ASV_111), one cnidarian (*Sarcophyton* ASV_9), one echinoderm (Echinodermata ASV_105), one mollusk (Gastropoda ASV_86), and five ASVs identified to kingdom level (Eukaryota ASV_30, ASV_54, ASV_78, ASV_164, and ASV_467) indicator taxa for the time factor. Eight indicator ASVs increased in abundance in 2017 (ASV_105, ASV_30, ASV_37, ASV_54, ASV_78, ASV_86, ASV_164, ASV_467) in 2017. And two (ASV_9 and ASV_11) in 2019.

### Environmental drivers shaping the spatio-temporal community patterns

**Figure 7:**
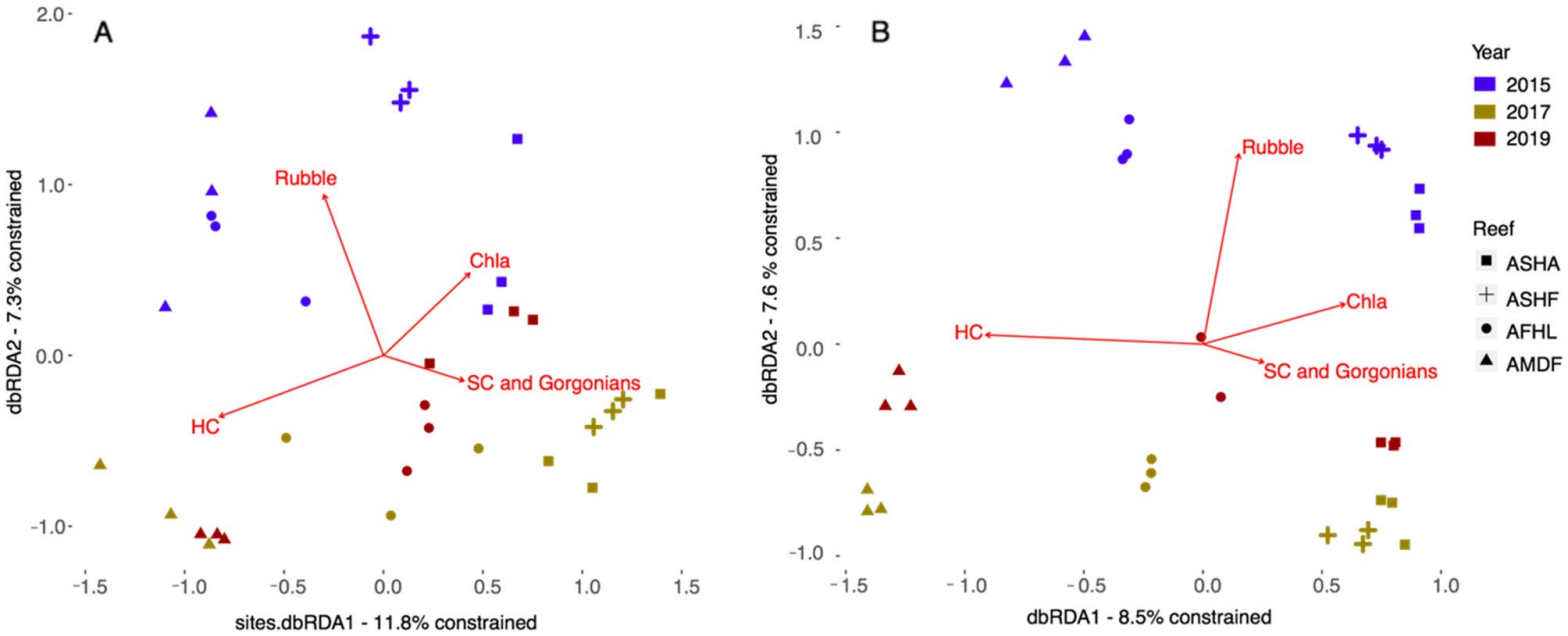
Distance-based redundancy analysis of the Bray–Curtis dissimilarity index for 2000 µm (**A**) and 106–2000 µm (**B**) fractions. each fraction. Shape of the symbol identifies the reef. Color shade of each symbol identifies the sampling date. Variation explained is constrained to the fourth non-correlated ecologically meaningful environmental variables selected. *ASHA* Abu Shoosha reef, *ASHF* Abu Shootaf, *AFHL* Al Fahal, *AMDF* Abu Madafi. *HC* Hard corals, *SC* Soft corals, *Chla* Chlorophyll-*a.*

For the > 2000 µm fraction the first axis of the distance-based redundancy analysis (dbRDA) explained 11.8% of the constrained variation with the cross-shelf gradient being evident along this axis especially for the 2017 and 2019 samples (Fig. 7A). Hard coral cover was associated with the first axis and particularly with the 2017 and 2019 Abu Madafi samples. The soft corals and gorgonians were also predominantly aligned along the first axis and were associated with the nearshore (Abu Shoosha) and midshore reefs (Al Fahal and Abu Shootaf) in 2017 and 2019. The second axis explained 7.3% of the constrained variation and separated the 2015 samples from those in 2017 and 2019. This axis was associated with the percentage of cover of rubble with the 2015 samples being positively linked. Chlorophyll-*a* concentrations (Chla) was associated with the nearshore reef Abu Shoosha in 2015 and 2019.

The cross-shelf pattern for the metabarcoding (106–2000 µm) fraction in the dbRDA is observable in the first axis of the dbRDA that explains 8.5% of the constrained variation (Fig. 7B). All the ARMS from the offshore reef Abu Madafi and ARMS from 2015 and 2017 were associated with increasing percentage cover of hard coral. Percentage cover of rubble was positioned almost parallel to the second axis and associated with the ARMS collected in 2015. The second axis separated the ARMS by time. Chla was mainly associated with nearshore reefs in 2015. Soft coral and gorgonians were associated with Abu Shoosha ARMS from 2017 and 2019 and Abu Shootaf ARMS from 2017.

## Discussion

To better understand the response of communities to long-term global pressures such as climate change, sustained studies utilizing standardized methods across spatial and temporal scales must be undertaken. Responses of coral reef communities to bleaching events have focused in its effects on coral communities globally^27,56,57^ and in the Red Sea^28,58^. Only a few studies focused on the largest fraction of the reef biodiversity (the cryptobenthos)^59,60^. While ARMS have previously been used to investigate differences in the cryptobiome across spatial gradients (e.g.,^5,16,48,49,51,53,54,61,62^, to the authors knowledge this is the first time a temporal aspect has been incorporated into the analysis of cryptobenthic communities associated with ARMS and across a bleaching event.

### Temporal variability: responses to the 2015/2016 mass bleaching event

Coral reefs present a highly variable cryptobiome community among reefs along cross shelf^16^, latitudinal gradients^5,17^, different habitats^54^, and seas^53^. This study shows that in addition to high variability across spatial scales, the cryptobiome is also highly variable through time. A shift in the benthic reef communities caused by the 2015 bleaching event^28,63,64^ could subsequently cause changes in the cryptobiome which relies on the reef 3D structure for their preferential ecological niches.

Our data covers the period immediately before the global mass bleaching event of 2015/2016 (June 2015) and four years after allowing the investigation of the effects of the bleaching event on the cryptobiome. While the central region of the Red Sea was not affected as strongly by the 2015/2016 bleaching event as it was by previous bleaching events^65^, changes in community composition were still apparent across the shelf gradient. Partitioning of the beta diversity showed that comparisons between pre-bleaching and post-bleaching samples resulted in higher turnover and lower nestedness of OTUs and ASVs than those between post-bleaching periods. This suggests a major shift after the bleaching event that was attenuated in the following years, which might indicate a recovery trend. The smaller organisms showed a slight return to the pre-bleaching community composition in 2019, yet far from signs of returning to pre-bleaching characteristics. The size of the communities and the associated length of life cycles (higher for fish) may bias the reaction time of reef-associated communities. Short lived species like those targeted by ARMS can, therefore, provide faster responses to disturbance events and be more informative in environmental impact assessment studies^7^. Despite signs of recovery from the effects of the 2015 bleaching event, full recovery may not happen quickly enough, if ever occur, if bleaching events become more frequent as expected^27^.

Annelida increased in relative abundance and percentage of number of reads after 2015 in the barcoding and metabarcoding fractions respectively, while Chordata decreased from 2015 to 2019 when assessed by metabarcoding. Annelids inhabit corals and non-living substrates^66^, however, in coral reefs most of species prefer areas not covered by living corals^67^. Regarding cryptobenthic fish, Froehlich, et al.^59^ observed a clear decline in all *Gobiodon* goby species in response to changes in their *Acropora* coral hosts after a sequence of cyclones followed by a bleaching event, with 78% of colonies uninhabited. Moreover, Bellwood et al.^60^ also reported that cryptobenthic fish communities may not recover to pre-bleaching characteristics even in cases of coral cover recovery, as shifts in coral communities may also persists beyond its cover. The close associations between cryptobenthic organisms and their coral hosts may magnify the shifts in community structure even if biodiversity increases^68^. Indeed, increase in biodiversity and density may not indicate better reef health as previous studies have found that number of species and abundance of cryptobenthic species tend to increase in coral rubble compared to live coral^67^. Also, regarding ocean warming, these small-sized organisms may have an advantage as increasing temperature may result in increasing growth rates, fostering successful settling and future survival^68^. In the smaller size fraction, there was a clear reduction in richness, measured as the number of ASVs, from 2015 to 2017 followed by an increase to levels similar to the pre-bleaching time. For the larger fraction we observed a steady increase in richness, measured as number of OTUs. Indeed, the shorter life span and higher sampled area to body size ratio of the smaller organisms allows us higher sensitivity to detect changes in reef communities.

### Cross-shelf patterns

Previous studies showed a cross-shelf gradient in both community composition and structure across the reefs analyzed here^16^. Pearman et al.^16^ described the offshore reefs in the Red Sea to account for the most unique OTUs, and observed distinct cryptobiome communities across the shelf. This gradient appears to be consistent across the temporal period studied here. This gradient is also consistent in other taxonomic groups including sessile benthic organisms and fish communities^69,70^. Thus, it appears that the bleaching event in 2015 did not cause a homogenization of the cryptobiome at the level that the shelf signature is lost. Individual coral reefs are physically isolated by kilometers of deeper, non-reef habitat, and often prevailing currents limits dispersal between reefs^71^, leading to dispersal limitation amongst the reefs^72^. Which could keep cryptobenthic communities between reefs distinct even when perturbed by extensive bleaching events or the associated increased in temperature. A high turnover in the cryptobiome amongst reefs is present in all time periods which was previously observed prior to the coral bleaching^16^ and is typical of other environments including soft bottom communities in the central Red Sea^73^ and in tropical coastal habitats^74,75^.

The cross-shelf distinction in coral reefs communities was clearer for smaller organisms. This is consistent with Soininen, et al.^76^ and Chust et al.^77^ who found that dispersal capabilities of species has a negative relationship with beta diversity. Indeed it has been shown that dispersal limitation had a greater effect on the cryptobiome in a pan-regional study compared to environmental conditions^53^. However, it should be noted that two different processing methodologies, as well as taxonomic level used in the comparisons (ASV and OTU), were undertaken for the different size fractions in this study and thus it cannot be ruled out that methodological biases could account for this finding. Future studies could involve the metabarcoding of the larger size fraction and the use of the same sequencing grouping methodology to minimize this bias as well as including other taxonomic groups such as bacteria and fungi.

Beta diversity is also driven by variations in local environmental conditions^78^ with the cryptobiome being shown to be affected by environmental differentiations^15,79^. Indeed, temperature, fishing pressure, nutrient levels, sedimentation, and pollution has been hypothesized to influence the communities of coral reefs^80,81^. The offshore reef, Abu Madafi, was associated with hard coral and algae, which were negatively correlated with SST while the other reefs were positively correlated with sea surface temperature. This suggests that the cryptobenthic community in the offshore reef is particularly vulnerable to future increases in temperature in agreement with Chaidez, et al.^82^. Indeed, Abu Madafi showed the largest reduction in species richness between 2015 and 2017 before partially recovering to 2015 levels in 2019.

The turnover increased exclusively after the bleaching event in the inshore reef Abu Shoosha, supporting our hypothesis. Indeed, the inshore reefs in the central Red Sea were strongly bleached compared to the midshelf and offshore reefs, particularly branching corals^28^. The type of community composition of the benthos indeed affects cryptobenthic communities^54^. Coker et al.^15^ reported that the branching corals *Acropora*, *Pocillopora*, and *Stylophora* influence community composition in the Cryptobenthos of the Red Sea. In our results, hard coral cover was related to samples from the offshore reef Abu Madafi and soft coral cover with samples from the inshore reef Abu Shoosha.

## Conclusions

Our results demonstrate highly dissimilar communities in the same reef complex refuting the neutral theory of biodiversity^83^ and showing what is expected for a community affected by environmental variation as seen in other coral reefs^84^. Even after the bleaching event, the nearshore to offshore gradient in cryptobenthic communities was still apparent, confirming out first hypothesis. Yet, responses were not always consistent through time across the shelf gradient.

The lack of sound knowledge on the natural variability of cryptobiota in the Red Sea hampers us from fully disentangling the effects of bleaching on the composition of these assemblages. Our results suggest that cryptobenthic communities will respond to bleaching events and that responses might be size-dependent. While for the largest organisms the number of OTUs increased after the bleaching event not returning to pre-bleaching, for the smaller fraction showed a clear decrease in the number of ASVs two years after the bleaching, with numbers recovering to pre-bleaching levels in 2019. Changes in community composition were also apparent between the response of organisms in both size fractions, reinforcing the importance of more comprehensive assessments of biodiversity patterns when investigating coral bleaching impacts. The partitioning of beta-diversity supported our hypothesis that temporal turnover would be higher in nearshore reefs, particularly in the larger size fraction. This study lays the foundation for future studies in spatio-temporal variability patterns of this critical component of the reef biodiversity using standardized quantifications, and in particular for comparisons with other regions more severely affected by the 2015/2016 global bleaching event, as the south-central Red Sea. However, a more in-depth analysis of shifts in coral species is also necessary as cryptic species often develop symbiotic or mutualistic relationships with specific corals and these might be disrupted even if the overall cover is maintained by other coral species. Directing our attention to the general changes in coral cover lacking deeper taxonomic resolution and disregarding the highly diverse cryptobiome, one might be overlooking relevant effects of bleaching on coral reef ecosystems processes and functioning. This is particularly alarming given the scarce knowledge we have about the ecological roles, life histories and distribution preferences of the members of the cryptobiome, limiting our ability to predict the resilience of coral reefs to climate change^68^.

## Methods

### Sampling design

Previous studies conducted in the Red Sea identified well-defined reef biodiversity cross-shelf patterns (nearshore to offshore reefs) of the cryptobiome^16^. Given the previously observed cross-shelf patterns, four reefs in the central Red Sea were selected under the framework of a long-term monitoring program in the Red Sea (two nearshore, one midshore, and one offshore). This framework allowed for the investigation of the components (turnover and nestedness) of beta diversity^85^. Turnover informs about the replacement of species between pairs of sites and nestedness about the prevalence of species^86^. It is important to know the contribution of turnover and nestedness to beta diversity in order to understand the ecological and historical processes that shaped the community studied^85^.

**Figure 8:**
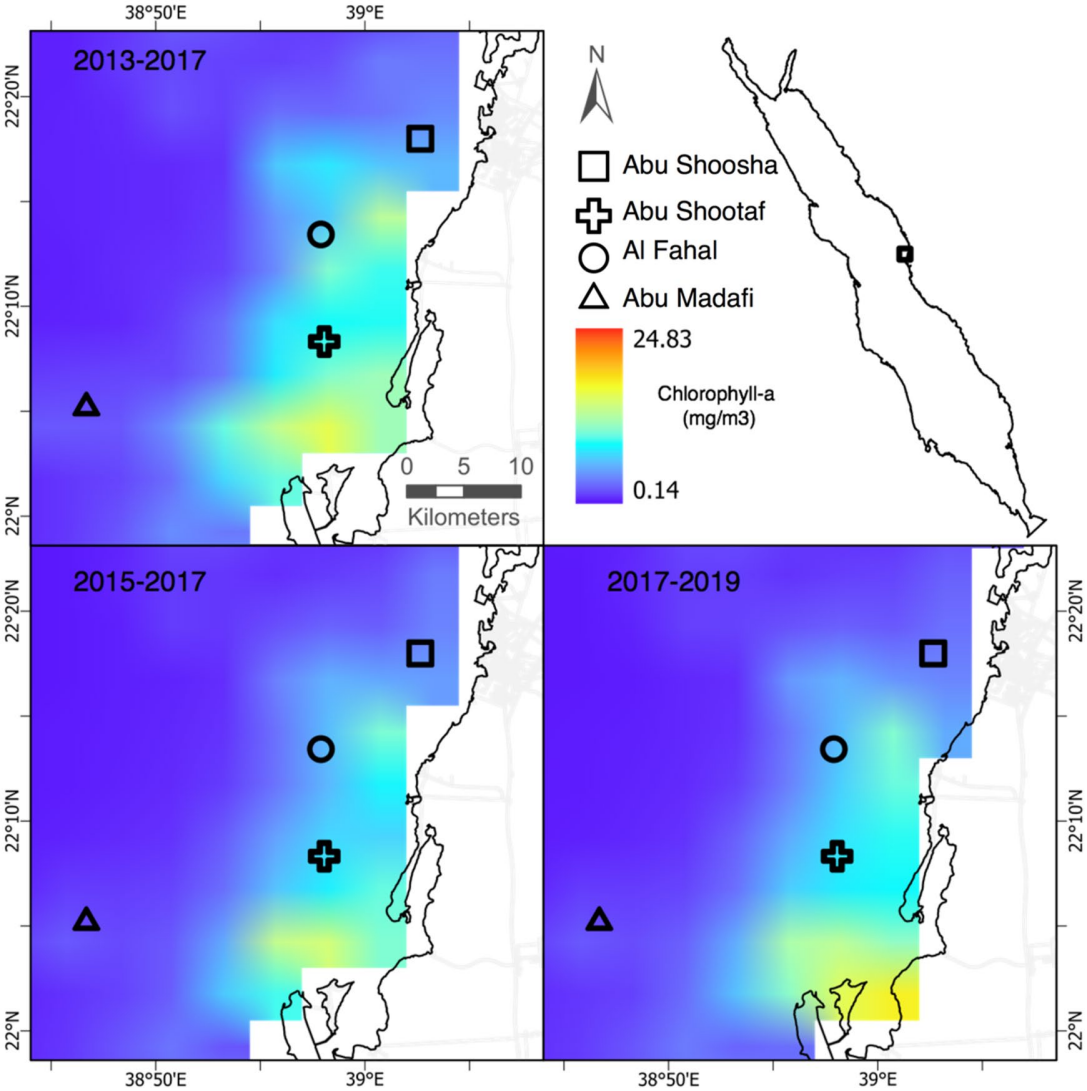
Reef sites sampled in the central East Red Sea coast (Saudi Arabia). Top right figure showing location of the sampling area in the Red Sea. Top left figure shows the average of Chlorophyll-*a* in mg/m^3^ (Chla) from June 2013 to May 2015. The bottom left, and bottom right figure shows the average for June 2015 to May 2017 and June 2017 to May 2019, respectively, of Chla in mg/m^3^. Maps were designed using ArcMap (Version 10.7.1.), Environmental Systems Research Institute, Inc., Redlands (esri.com) using data from NASA MODIS 4 km resolution.

A total of 33 ARMS units were deployed and retrieved from three sampling periods. Triplicate ARMS were placed in four reefs located across the Saudi Arabian coastal shelf (sites were considered nearshore, midshore, and offshore given their relative distance from shore) in the central Red Sea starting in 2013 (Fig. 8), and replaced and processed every two years. The retrieval dates occurred in May 2015, May 2017, and 2019. Data from the samples retrieved in 2015 were already published in^16^. However, in this study we reanalyze the 2015 data together with new data from 2017 and 2019 to assess temporal changes. Due to a logistical restriction, the retrieval in 2019 collected two reefs in June (Abu Shoosha and Al Fahal) and one in November (Abu Madafi). Locations, deployment and retrieval dates are provided in Table S-4. Abu Shoosha was considered nearshore, Abu Shootaf and Al Fahal midshore, and Abu Madafi offshore following the classification of Pearman et al.^16^. The first sampling date in 2015 occurred prior to the bleaching event in the central Red Sea, which was recorded from September 2015, with nearshore reefs suffered a higher percentage of bleached corals than midshore and offshore reefs^28^.

### Environmental characterization

Sea surface temperature (SST), chlorophyll-*a* concentrations, (Chla) and percentage cover of ecologically meaningful groups (see "Reef surveys" section below) from benthic reef surveys were included as explanatory variables for further analysis of the cryptobiome. Environmental variables showed a skewed distribution and were transformed using Box Cox, to minimize the skewed nature. To assess correlations between the environmental variables, a Pearson R correlation was used on the normalized and transformed environmental variables^87^. Variables with significant (*p* < 0.01) results greater than 0.5 or less than − 0.5 were assessed and only the environmental variables rubble, hard coral, soft corals and gorgonians, and Chla were retained.

### Remote sensing data

Eight-day averages of nighttime SST and daily averages of Chla were downloaded from NASA Oceancolor website https://oceandata.sci.gsfc.nasa.gov/opendap/catalog.xml on 25th December 2020 choosing MODIS Aqua with 4 km resolution. The time interval of the deployment period was retrieved for each sample unit.

### Reef surveys

Coral reef benthic communities were assessed based on triplicate (separated by 5 m) 20 m length by 1 m wide photo-transects conducted at each sampling location during the recovery of the ARMS unit between 8 and 10 m depth. Photo quadrats of 1 × 1 m were taken every two meters as in Pearman et al.^47^. The ecological groups and the major benthic components (hard coral, soft coral and gorgonians, crustose coralline algae, other algae, turf algae, pavement or rock, rubble, and sand) were identified and percent cover estimated in Coral Point Count with Excel extensions for 48 randomly distributed points as described in Kohler and Gill^88^.

### Deployment, recovery, and processing of ARMS

ARMS were deployed by SCUBA at approximately 10 m depth on the hard reef framework. SCUBA was used to collect the ARMS units; a collection container with 106 µm mesh drainage holes was placed over each unit to retain any mobile organisms inhabiting the units. Once on board the boat, units were placed in plastic containers and submerged with filtered (106 µm) seawater from the site.

To disassemble the ARMS, each plate was brushed inside the filtered sea water (106 µm), to remove mobile organisms, then scraped to remove the incrusting and sessile organisms. Sea water was sieved through 106 µm, 500 µm, and 2000 µm mesh to divide the mobile organisms into three fractions: (i) 106–500 µm, (ii) 500–2000 µm, and (iii) > 2000 µm. Each fraction was then homogenized in a blender and preserved in 96% ethanol.

The two smallest fractions and the scraped sessile fraction were processed following a metabarcoding technique. The organisms of the larger > 2000 µm were individually segregated and morphologically identified to its lowest possible taxonomical group before DNA barcoding.

### Ethics declarations

This research followed the guidelines for sampling recognized at King Abdullah University of Science and Technology (KAUST) at the start of the study. The research permits for sampling in Saudi Arabian territorial seas were procured from the Saudi Arabian coastguard. This study did not target protected or endangered species; therefore, it did not require special authorization. At the time of sampling, no governing body of ethics for animal research was established in KAUST or Saudi Arabia. Consequently, we could not attain ethics approval or waiver.

### DNA extraction, amplification, and sequencing

#### Barcoding

A tissue sample was taken from organisms of the > 2000 µm fraction and DNA extracted using the Qiagen DNeasy DNA extraction kit, following manufacturers protocol. To accelerate the DNA extraction process of the specimens, tissue was sampled at an approximate size of 1 mm^3^ instead of weighing each individual tissue sample. Smaller specimens, which did not have the required tissue size, were placed in the lysis buffer whole. A region of 658 bp of the mitochondrial cytochrome oxidase I (COI) gene was amplified using the primer combination jgLCO1490 (TITCIACIAAYCAYAARGAYATTGG) and jgHCO2198 (TAIACYTCIGGRTGICCRAARAAYCA)^89^. A polymerase chain reaction (PCR) was done using 10 μl of GoTaq G2 Hot Start Master Mix (Promega), 0.6 μl of each primer at 10 μM, 0.2 μl of 20 mg mL^-1^ bovine serum albumin (BSA), and 1 μl of extracted DNA for a total of 19 μl of reaction for the samples of retrieved in 2015 and 2017. The thermocycling profile of the 2015 and 2017 samples consisted of an initial denaturation step at 95 °C for 5 min followed by 4 cycles of 94 °C-30 s, 50 °C-45 s, and 72 °C-1 min, and by 34 cycles of 94 °C-30 s, 45 °C-45 s and 72 °C-1 min, and a final 8 min elongation phase at 72 °C. Samples from 2019 were amplified in the same region with the same primers using 12.5 μl of QIAGEN Multiplex PCR master mix, 2.5 μl primer at 0.2 μM, 9 μl of RNase-free water, and 1 μl of extracted DNA for a total of 25 μl of reaction. Following the thermocycling profile consisting of an initial denaturation step at 95 °C for 15 min followed by 4 cycles of 94 °C-30 s, 50 °C-1 min 30 s, and 72 °C-1 min, and by 34 cycles of 94 °C-30 s, 45 °C-1 min 30 s and 72 °C-1 min, and a final 10 min elongation phase at 72 °C. PCR products were inspected on 1.5% agarose gels stained with 4 μl of SYBR™ Safe DNA gel stain per 100 mL. Successful PCR products were purified applying 2 μl of Illustra™ ExoProStar™ 1-step from GE Healthcare to 8 μl of PCR product. The PCR product was sequenced in Sanger ABI 3730 capillary platform using 5 μl of primer at 20 pmol and 10 μl of purified PCR product at the King Abdullah University of Science and Technology (KAUST) Bioscience Core Laboratory (BCL).

#### Metabarcoding

For the two smaller size fractions of the bulk mobile organisms 10 g of material was used as an input for the Powermax Soil DNA kit (MO BIO). Extractions were undertaken as per the manufacturer’s instructions with the exception of the bead-beating step. This step was replaced by shaking incubation overnight at 56 °C with the addition of Proteinase K (0.4 mg/mL). DNA was amplified using a versatile primer set amplifying a 313 bp fragment of the COI gene (Forward: GGWACWGGWTGAACWGTWTAYCCYCC; Reverse: TAIACYTCIGGRTGICCRAARAAYCA^89^). For amplification, the PCR conditions were an initial 3 min denaturation step at 98 °C, followed by 27 cycles at 98 °C for 10 s, 46 °C for 45 s and, 72 °C for 45 s, with a final extension of 5 min at 72 °C. All PCR reactions were done in triplicate, using 0.4 μl of 10 μM primers, 10 μl of Phusion High Fidelity Mix (2X), 7.2 μl of water and 2 μl (~ 10 ng) of DNA. PCR triplicates were combined and 20 μl of the combined PCR products were cleaned and normalized using SequelPrep Normalization plates (ThermoFisher Scientific) resulting in a final concentration of ~ 1 ng/μl. To add Illumina Nextera tags, a second round of PCR amplification of 8 cycles using KAPA 2 × HiFi Hot Start ReadyMix was undertaken following the manufacturer’s recommendations. The SequelPrep Normalization plates (ThermoFisher Scientific) were used to undertake a second round of cleaning and normalization. Sequencing (2 × 300 bp) was done on an Illumina MiSeq sequencing platform (v3 chemistry) at the King Abdullah University of Science and Technology (KAUST) Bioscience Core Laboratory (BCL), using a spike of 10% PhiX. Raw reads were deposited at the NCBI Short Red Archive under the project accession (To be deposited).

#### Bioinformatics

##### Barcoding

Two directional sequences were trimmed from the 5’ and 3’ end during assembly, when chance of error was higher than 5%. Assemblies were reviewed for stop codons and frame shifts in Genious (Biomatters). Consensus sequences of the assemblies were aligned and primer sequences were removed. A Bayesian clustering algorithm with lower 3 and upper 4 variance interval was used to define OTUs with a minBoot threshold of 51^90^. Taxonomy of the sequences were assigned comparing the representative sequences of each OTU obtained from the Bayesian clustering algorithm to morphological identifications done with the organisms’ photograph taken before fixation. Specimens that failed to be sequenced were added to an OTU if the morphology observed in their photograph matched the taxonomy or the morphology of the representative organisms of an OTU. OTUs that were not identified morphologically to species level were matched to the NCBI public databases using blastn algorithm and assigning a species hit to sequences similarity higher than 98%.

#### Metabarcoding – inference of amplicon sequence variant (ASV) and their taxonomic assignments

Raw sequences were automatically demultiplexed on the MiSeq machine. Primers were trimmed from the sequences using cutadapt with a maximum of one mismatch allowed (parameters: -e 0.05 –discard-untrimmed). The DADA2 package^91^ within R^92^ was used for the processing of the reads. Briefly reads were truncated 165 and 160 bp for forward and reverse reads respectively) and filtered with a maximum allowable number of “expected errors” (maxEE) of four (forward reads) and six (reverse reads). Sequences were dereplicated and sequence variants were inferred based on a parametric error matrix constructed from the first 10^8^ bp of the sequences. Singletons were discarded and the remaining paired-end reads merged with a minimum overlap of 10 bp and no mismatches in the overlap allowed. Sequences that were shorter than 312 bp or longer than 314 bp were removed from analysis before chimeric sequences were removed using the removeBimeraDenovo script within DADA2. Pseudogenes were detected and removed using Multiple Alignment of Coding Sequences (MACSE^93^) against the MIDORI database^94^ as described in Leray and Knowlton Leray and Knowlton^54^. Firstly, sequences were translated and aligned using the invertebrate code 5 and then the vertebrate code 2. Any sequences containing a stop codon or possessing greater than two frame shifts were considered as pseudogenes and removed from further analysis. Samples were subsampled to evenly for downstream comparison. Taxonomy was assigned against both the NCBI and BOLD databases using RDP classifier algorithm in dada2 with a minBoot threshold of 51^95^. The two smaller mobile fractions were combined and used in further analysis for simplification. The sessile fraction is not used in this publication.

### Data analysis

#### Analysis of diversity

Alpha diversity (observed OTUs and ASVs for all fractions, and Shannon diversity and abundance for the barcoded organisms) was assessed using the non-parametric test Kruskal–Wallis, due to the skewed nature of the data, for the factors reef and time. The number of unique and shared species among reefs and time was visualized in Venn diagrams plotted in VennDiagram package of R^96^ and figures edited in Graphic (Picta). Access to sites in Saudi Arabian waters are subject to approvals from government authorities; such approvals may change on short notice due to government activities on the nearby coastal areas. In 2019, we were not able to access Abu Shootaf because of permitting issues. Therefore, the reef Abu Shootaf was removed from the univariate analysis, given the lack of 2019 samples from this reef. However, in the Venn diagram categorized by time we kept Abu Shootaf. Composition plots for each reef and time were plotted at order level using phyloseq in R and ggplot2^97,98^.

#### Patterns of community structure across a shelf gradient and time

A permutational multivariate analysis of variance (PERMANOVA) was done using the Bray Curtis and Jaccard dissimilarity index distance matrix for the factors reef and time with an interaction term as well using vegan package in R^99^. PostHoc test with Bonferroni adjustment of probability was conducted for the Bray Curtis and Jaccard dissimilarity index distance matrix for reef and time factors using RVAideMemoire program in R^100^. Bar plots were done in phyloseq and ggplot2 to visualize composition in the order level for each sample^97,98^.

The indicator taxa were found using the command multipatt of indicspecies in R^101^. We used 299 iterations of the indval value and retained the taxa that were consistently selected (> 90% of the times). We used 999 permutations in each iteration and restrict the permutation within the year for the reefs and within the reef for the years. Kruskal Wallis test was performed on the abundances of the barcoding fraction for the selected indicator taxa. Kruskal Wallis was chosen over ANOVA given that our data did not meet the ANOVA assumption of homogeneity of variance. No statistical analysis was done in the number of reads of the selected taxa for the metabarcoding fraction, because the error in measuring abundance in a metabarcoding protocol would be higher than the barcoding and a Kruskal Wallis test would provide low statistical power.

#### Environmental influences in community structure between reefs through time

A distance-based redundancy analysis (dbRDA) was performed to visualize the direction and extent of the associations between environmental factors and community structure. The dbRDA was performed for Bray–Curtis and Jaccard dissimilarity distance matrices^102^. Biological data was transformed using the Hellinger method in the vegan package to give greater importance to the OTUs which were not dominant^5^.

#### Beta diversity analyses

A principal coordinate analysis (PCO) was used to visualized the differences in the composition of the cryptobiome between ARMS. The PCO was done in the package labdvs of R^103^ using the Jaccard dissimilarity metric. The nestedness and turnover was assessed between pairs of ARMS of different sampling times for each reef and between reefs for each sampling time using the Baselga Jaccard method in the adespatial package of R^85,104^.The nestedness and turnover comparisons were visualized in boxplots. Differences in nestedness and turnover amongst sampling time and reefs were tested with a one-way ANOVA for those that were normally distributed and had homogeneity of variance. Otherwise, a Kruskal–Wallis test was used.

## Supplementary Information

Supplementary Information 1.

Supplementary Information 2.

## Data Availability

Raw reads will be deposited at the NCBI Short Red Archive prior to publication.
