# Supplementary material for: Inter-annual variability patterns of reef cryptobiota in the central Red Sea across a shelf gradient
Source: Sci Rep. 2022 Oct 9;12:16944. doi: 10.1038/s41598-022-21304-2 (PMC9548503; doi:10.1038/s41598-022-21304-2)
Supplement: Supplementary file 1 — Supplementary Information 1. [file 41598_2022_21304_MOESM1_ESM.pdf]

Inter-annual variability patterns of reef cryptobiota in the central Red Sea across a shelf gradient

Villalobos, R.[1], Aylagas, E.[1,2], Pearman, J.K.[3], Curdia, J.[1], Lozano-Cortés, D.[4], Coker, D.J.[1], Jones, B., Berumen, M.L.[1], & *Carvalho, S.[1]

[1]King Abdullah University of Science and Technology (KAUST), Red Sea Research Center, Thuwal 23955-6900, Saudi Arabia
[2]The Red Sea Development Company, 5th Floor, MU04 Tower, ITCC Complex, AlRaidah Digital City, Al Nakhil District 3807, Riyadh 12382 – 6726, Saudi Arabia
[3]Coastal and Freshwater Group, Cawthron Institute, Nelson, New Zealand
[4]Environmental Protection, Saudi Aramco, Dhahran, Saudi Arabia
*corresponding authors' email: susana.carvalho@kaust.edu.sa

Table S-1. Alpha diversity in the cross-shelf and between sampling times. Kruskal-Wallis analysis of the number of ASVs of the 106-2000 µm fraction and number of OTUs, the Shannon diversity index, and the abundance of the >2000 µm fraction for factors time and region.

	Time		Reef	
	Chi Square	p	Chi Square	p
Number of ASV Mobile	7.8	**0.02**	1.7	0.42
Number of OTU 2000 µm	6.7	**0.04**	4.8	0.09
Shannon 2000 µm	5.8	0.05	3.9	0.15
Abundance 2000 µm	10.2	**<0.01**	3.0	0.22

Table S-2. Results of the permutational multivariate analysis of variance in the Bray-Curtis and Jaccard dissimilarity matrices for the >2000 μm and the 106-2000 μm fractions between samples using time and reef as factors.

Fraction	Distance Matrix	Factor	MeanSqs	F.Model	Pr(>F)
>2000	Bray-Curtis	Time	0.73	1.91	0.001
		Reef	0.73	2.31	0.001
		Time:Year	0.62	2.33	0.001
	Jaccard	Time	0.70	1.99	0.001
		Reef	0.67	1.90	0.001
		Time:Year	0.61	1.73	0.001
106-2000 *μ*m	Bray-Curtis	Time	0.71	1.74	0.003
		Reef	0.81	2.14	0.001
		Time:Year	0.53	1.53	0.001
	Jaccard	Time	0.63	1.54	0.001
		Reef	0.69	1.68	0.001
		Time:Year	0.53	1.29	0.001

Table S-3. Results of the post hoc analysis of the permutational multivariate analysis of variance in the Bray-Curtis and Jaccard dissimilarity matrices for the >2000 µm and the 106-2000 µm.

106-2000 µm	Bray-Curtis		AFHL	AMDF	ASHA
		AMDF	0.006	-	-
		ASHA	0.006	0.006	-
		ASHF	0.006	0.012	0.03
			2015	2017	
		2017	0.003	-	
		2019	0.003	0.033	
	Jaccard		AFHL	AMDF	ASHA
		AMDF	0.006	-	-
		ASHA	0.006	0.006	-
		ASHF	0.006	0.006	0.012
			2015	2017	
		2017	0.003	-	
		2019	0.003	0.063	
>2000	Bray-Curtis		AFHL	AMDF	ASHA
		AMDF	0.018	-	
		ASHA	0.054	0.006	-
		ASHF	0.006	0.006	0.81
			2015	2017	
		2017	0.003	-	
		2019	0.003	0.021	
	Jaccard		AFHL	AMDF	ASHA
		AMDF	0.018	-	-
		ASHA	0.072	0.006	-
		ASHF	0.012	0.006	0.57
			2015	2017	
		2017	0.003	-	
		2019	0.003	0.021	

Table S-4. Location and dates of the sampling sites.

Reef	Short name	Deployment	Retrieval	Latitude N	Longitude E
Abu Shoosha	ASHA	May 2013	May 2015	22.299799	39.044185
Abu Shoosha	ASHA	May 2015	July 2017	22.299799	39.044185
Abu Shoosha	ASHA	July 2017	May 2019	22.299799	39.044185
Abu Shootaf	ASHF	May 2013	May 2015	22.138715	38.967578
Abu Shootaf	ASHF	May 2015	July 2017	22.138715	38.967578
Al Fahal	AFHL	May 2013	May 2015	22.223781	38.964983
Al Fahal	AFHL	May 2015	July 2017	22.223781	38.964983
Al Fahal	AFHL	July 2017	June 2019	22.223781	38.964983
Abu Madafi	AMDF	May 2013	May 2015	22.089319	38.778075
Abu Madafi	AMDF	May 2015	July 2017	22.089319	38.778075
Abu Madafi	AMDF	July 2017	November 2019	22.089319	38.778075

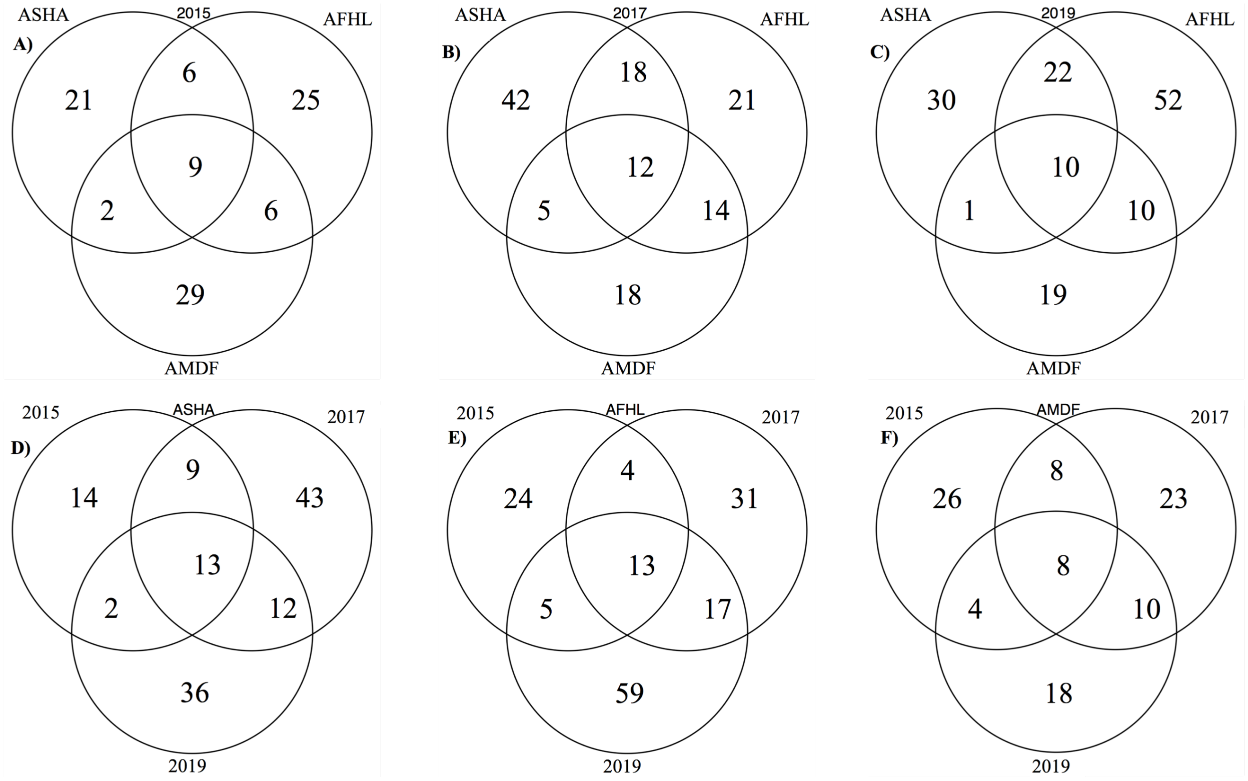

Figure S-1. - Shared and unique OTU of each reef through the 3 sampling times (A-C) and between reefs at each sampling time (D-F) for the barcoding fraction.

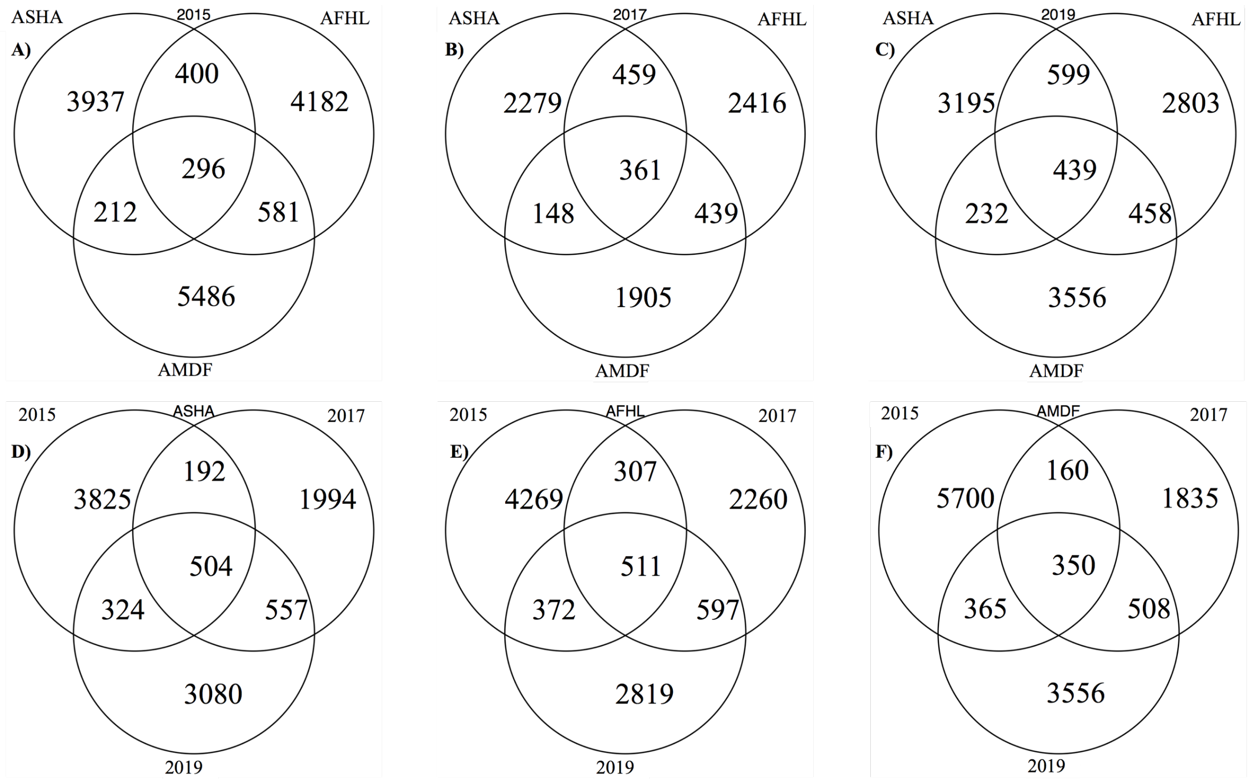

Figure S-2. - Shared and unique OTU of each reef through the 3 sampling times (A-C) and between reefs at each sampling time (D-F) for the metabarcoding fraction.
